# From Bedside to the Bench—A Call for Novel Approaches to Prognostic Evaluation and Treatment of Empyema

**DOI:** 10.3389/fphar.2021.806393

**Published:** 2022-01-20

**Authors:** Sophia Karandashova, Galina Florova, Steven Idell, Andrey A. Komissarov

**Affiliations:** ^1^ Department of Pediatrics, University of California San Francisco, San Francisco, CA, United States; ^2^ Department of Cellular and Molecular Biology, The University of Texas Health Science Center at Tyler, Tyler, TX, United States

**Keywords:** empyema, fibrinolytic therapy, preclinical, treatment, animal model

## Abstract

Empyema, a severe complication of pneumonia, trauma, and surgery is characterized by fibrinopurulent effusions and loculations that can result in lung restriction and resistance to drainage. For decades, efforts have been focused on finding a universal treatment that could be applied to all patients with practice recommendations varying between intrapleural fibrinolytic therapy (IPFT) and surgical drainage. However, despite medical advances, the incidence of empyema has increased, suggesting a gap in our understanding of the pathophysiology of this disease and insufficient crosstalk between clinical practice and preclinical research, which slows the development of innovative, personalized therapies. The recent trend towards less invasive treatments in advanced stage empyema opens new opportunities for pharmacological interventions. Its remarkable efficacy in pediatric empyema makes IPFT the first line treatment. Unfortunately, treatment approaches used in pediatrics cannot be extrapolated to empyema in adults, where there is a high level of failure in IPFT when treating advanced stage disease. The risk of bleeding complications and lack of effective low dose IPFT for patients with contraindications to surgery (up to 30%) promote a debate regarding the choice of fibrinolysin, its dosage and schedule. These challenges, which together with a lack of point of care diagnostics to personalize treatment of empyema, contribute to high (up to 20%) mortality in empyema in adults and should be addressed preclinically using validated animal models. Modern preclinical studies are delivering innovative solutions for evaluation and treatment of empyema in clinical practice: low dose, targeted treatments, novel biomarkers to predict IPFT success or failure, novel delivery methods such as encapsulating fibrinolysin in echogenic liposomal carriers to increase the half-life of plasminogen activator. Translational research focused on understanding the pathophysiological mechanisms that control 1) the transition from acute to advanced-stage, chronic empyema, and 2) differences in outcomes of IPFT between pediatric and adult patients, will identify new molecular targets in empyema. We believe that seamless bidirectional communication between those working at the bedside and the bench would result in novel personalized approaches to improve pharmacological treatment outcomes, thus widening the window for use of IPFT in adult patients with advanced stage empyema.

## Using Minimally Invasive Treatments for More Advanced-Stage Empyema: A Novel Approach

Empyema is a common, potentially life-threatening infection of the pleural space with several different etiologies (Franklin et al., 2021). The incidence and mortality of empyema appears to be increasing over the past 4 decades despite advances in modern medicine (Finley et al., 2008; Bender et al., 2009; Burgos et al., 2011; Grijalva et al., 2011; Burgos et al., 2013; Bedawi et al., 2018; Godfrey et al., 2019). The estimated sixty-five thousand cases of empyema that occur annually in the United States demonstrate a mortality between 10 and 27%, with greater likelihood of worse outcomes if initial treatment is unsuccessful (Chapman and Davies, 2004; Maskell et al., 2006; Porcel and Light, 2006; Koegelenberg et al., 2008; Ben-Or et al., 2011; Light, 2011; Abu-Daff et al., 2013; Reichert et al., 2017; Shen et al., 2017; Meyer et al., 2018; Semenkovich et al., 2018). Treatment of patients with empyema has been estimated to cost up to half a billion dollars per annum (Taylor and Kozower, 2012). The American Thoracic Society recognizes three sequential stages in the development of empyema (I) early exudative, (II) fibrinopurulent, and (III) organized. Although evacuation of pleural fluid and lung re-expansion can be achieved by thoracentesis in stage I, the increased fibrin deposition in the pleural space observed in stage II may require treatment with intrapleural fibrinolytic therapy (IPFT) or video-assisted thoracoscopic surgery (VATS). Treatment of patients with stage II empyema remains controversial in adults, with pharmacological and surgical approaches being used contingent on practitioner preference and hospital protocols. Stage III, which features a thick pleural rind and trapped lung, often requires open decortication of the lung via thoracotomy (Andrews, 1965; Light, 2011; Søgaard et al., 2014; Reichert et al., 2017; Reichert et al., 2018b; Reichert and Bodner, 2018; Semenkovich et al., 2018; Godfrey et al., 2019). The presence of pleural thickening on chest computed tomography (CT) coupled with fever could indicate severe, organizing empyema (Stefani et al., 2013). Patients with empyema complicated by lung abscess have a higher risk of failure with surgical intervention and a higher rate of mortality (23% compared to 14%) (Huang et al., 2010). Notably, VATS was recently shown to be effective in treatment of stage III empyema in adult patients (Hajjar et al., 2016; Aljehani and Alabkary, 2018; Reichert et al., 2018a; Reichert et al., 2018b; Semenkovich et al., 2018), where thoracotomy with decortication of visceral pleura had previously dominated. Thus, the shift from a more invasive surgical approach for stage III empyema towards VATS becomes feasible. A trend towards less invasive procedures to treat stage II/III empyema in combination with the recently reported equal efficacy of VATS and IPFT (Ohara et al., 2018; Samancilar et al., 2018; Kermenli and Azar, 2021) opens a window of opportunity to expand the use of pharmacological treatment in advanced stage empyema.

## Treatment of Pediatric Empyema With Minimal Mortality: An Example of the Trend

IPFT is particularly effective in pediatric patients with empyema (de Benedictis et al., 2000; Thomson et al., 2002; Barbato et al., 2003; Barnes et al., 2005; Sonnappa et al., 2006; Sonnappa and Jaffe, 2007; Bianchini et al., 2010; Stefanutti et al., 2010; Abu-Daff et al., 2013; Israel and Blackmer, 2014; Nie et al., 2014; Coelho et al., 2016; James et al., 2017; Livingston et al., 2017). In sharp contrast to adults, retrospective studies of VATS in pediatric patients with empyema (Avansino et al., 2005; Chibuk et al., 2011; Cohen et al., 2012; Cohen et al., 2013), showed minimal mortality (n = 101) (Chen et al., 2009). While there is still a debate regarding whether VATS is more expensive than IPFT (Sonnappa et al., 2006; Cohen et al., 2008; St Peter et al., 2009) or not (Shah et al., 2010; Derderian et al., 2020), the latter is advocated for by some as the preferred initial treatment of empyema in pediatric patients (Livingston et al., 2016b; Derderian et al., 2020). IPFT in pediatric practice is remarkably effective but still empiric. IPFT with single chain tissue-type plasminogen activator (tPA, Alteplase) was more effective than saline irrigation (Hanson et al., 2015). Meta-analysis (Pacilli and Nataraja, 2018) and multiple studies have demonstrated equivalent efficacy between VATS and IPFT with urokinase (uPA) (Sonnappa et al., 2006; Marhuenda et al., 2014; Sonnappa, 2015; Pacilli and Nataraja, 2018) or tPA (St Peter et al., 2009; Livingston et al., 2016b) in treatment of empyema in pediatric patients. As a result, IPFT is recommended as the first line treatment for empyema in pediatrics (St Peter et al., 2009; Kobr et al., 2010; Stefanutti et al., 2010; Slaats et al., 2019; Baram and Yaldo, 2020; Livingston et al., 2020). Further studies of treatment with tPA (4 mg) alone or with DNase (Dornase; 5 mg) in empyema in pediatric patients (n = 97; sequential injection, 1 h apart) demonstrated that DNase does not improve the efficacy of tPA (Livingston et al., 2017; Livingston et al., 2020). While there are gaps in our understanding, the differences between empyema in adults and children, including a low mortality in the latter favors applying minimally invasive pharmacological treatment as the first option. Preclinical studies may further assist in closing this gap in our knowledge of age-dependent progression of empyema.

## Treatment of Advanced Stage Empyema in Adults Cannot be Extrapolated From Pediatric Research

Multiple recent clinical trials have demonstrated the efficacy of IPFT in adult patients with empyema (Thommi et al., 2007; Rahman et al., 2011; Thommi et al., 2012; Popowicz et al., 2014a; Popowicz et al., 2014b; Nie et al., 2014; Piccolo et al., 2014; Piccolo et al., 2015; Barthwal et al., 2016; Majid et al., 2016; Mehta et al., 2016; Vial et al., 2016; Bishwakarma et al., 2017; Popowicz et al., 2017; Beckert et al., 2019; Godfrey et al., 2019). However, the strikingly higher mortality in adults does not allow for extrapolation of treatment strategies used in pediatrics and speaks to the imperative for preclinical studies focused on the mechanisms governing development and resolution of empyema in adults. Advanced stage empyema is considered a risk factor for failure of IPFT (Himelman and Callen, 1986); (Moulton et al., 1995; Temes et al., 1996). Patients that present with extensive loculation, lung abscess, and pleural thickening are more likely to fail IPFT (Davies et al., 1999; Thommi et al., 2007; Chen et al., 2016; Khemasuwan et al., 2018) and require surgical intervention (Davies et al., 1999; Maskell et al., 2005; Thommi et al., 2007). On the other hand, a randomized, double blinded trial demonstrated that Alteplase (25 mg/100 ml, 1 h clamp, suction after second hour, daily up to 3 days) was both more effective than placebo (95 and 12%, respectively) and safe (Thommi et al., 2012). While meta-analyses also have revealed the advantages of IPFT over placebo there is still controversy regarding what constitutes optimal therapy (Janda and Swiston, 2012). Thus, in contrast to pediatric practice, there is insufficient data to recommend routine use of IPFT in adults. Notably, a meta-analysis did not find a statistically significant difference in mortality between surgical and non-surgical management of pleural empyema, with VATS potentially associated with a shorter hospital stay (Kwon, 2014; Redden et al., 2017; Chong et al., 2021). High-risk subsets of adults with empyema, such as pregnant patients, often have markedly limited data available regarding the safety and efficacy of IPFT. Systemic fibrinolytics have been used in pregnant patients with life-threatening or potentially debilitating conditions such as ischemic stroke, myocardial infarction, and venous thrombotic events, with no apparent increase in complications from that observed in non-pregnant adults (Leonhardt et al., 2006). The few publications available concur that likewise, IPFT is effective and can be safely used in pregnancy, but should be used on a case-by-case basis given the lack of clinical trials and limited data regarding possible fetal complications (Torbic et al., 2017; Dikensoy et al., 2018; Amariei et al., 2019; Nasralla et al., 2021). Thus, we propose that preclinical studies with appropriate animal models can inform the limits of IPFT and enhance the design of clinical trials aimed to improve the treatment of advanced stage empyema in adults.

## The MIST2 Protocol: Attempts at Revision Reflect the Failure of a “One Dose for All” Concept

Over the past decade, cost effective (Asciak et al., 2019) IPFT with a combination of tPA and deoxyribonuclease (DNase) (Dornase), as demonstrated in the Multicenter Intrapleural Sepsis Trial 2 (MIST2) (Rahman et al., 2011), has been broadly embraced as IPFT for empyema (Abu-Daff et al., 2013; Popowicz et al., 2014a; Popowicz et al., 2014b; Israel and Blackmer, 2014; Piccolo et al., 2014; Piccolo et al., 2015; Majid et al., 2016; Bishwakarma et al., 2017; Majid et al., 2017; Popowicz et al., 2017; Innabi et al., 2018; Kheir et al., 2018; Fitzgerald et al., 2019; Fitzgerald et al., 2021). A recent expert consensus statement (Chaddha et al., 2021) described the level of severity of empyema that should be treated with tPA/DNase injected concurrently, appropriate dosing (10/5 mg, respectively), dwelling time, dose schedule (twice a day), and an algorithm for IPFT, thus re-establishing a universal “one for all” treatment approach to empyema based on the MIST2 protocol. On the other hand, there have been multiple calls for optimization of the MIST2 strategy (Chan et al., 2018) by adjusting the doses of tPA and DNase (Innabi et al., 2018; Hart et al., 2019), dosing schedule (Majid et al., 2016; Mehta et al., 2016; Majid et al., 2017), and the use of plasminogen activators other than tPA (Alemán et al., 2015; Beckert et al., 2019; Bédat et al., 2019), which may indicate growing concerns regarding MIST2 protocol-based IPFT among clinicians. Dose de-escalation studies in patients in Australia were designed to determine a “minimal effective dose” of the drug in humans, with the goal of decreasing side effects such as bleeding (Popowicz et al., 2017). Other approaches were assessed in an attempt to increase the safety and efficacy of the MIST2 (Rahman et al., 2011) protocol, including increasing the dose of DNase with a concurrent decrease in tPA (Innabi et al., 2018), increasing dosing intervals (once a day Alteplase/DNase for 3 days, n = 55; 92.7% success) (Mehta et al., 2016), the use of tPA alone (Majid et al., 2016), and the administration of Alteplase and DNase concurrently (Majid et al., 2017). A 5-year analysis of complex pleural fluid collections (n = 103; 82 empyema) treated with Alteplase alone (6 mg daily) demonstrated the high efficacy of this treatment (need for surgery 7.3%, mortality 17.1%) (Heimes et al., 2017). A second 5-year observational study reported 83% efficacy for Alteplase in complicated pleural effusions (CPE) and 62.7% in empyema (n = 88 and 14, respectively) (Barthwal et al., 2016). Alteplase alone in doses between 10 and 100 mg daily (n = 120) was successful in 85% of empyema cases (Thommi et al., 2007). However, even high doses of Alteplase often failed in advanced stage empyema (Thommi et al., 2007). These reports indicate uncertainty in the clinical field regarding the optimal treatment of empyema in adults, invalidating the current concept that one dose can treat everyone and supporting further studies on a personalized approach to pharmacological management of empyema in adults. Thus, preclinical studies with validated models of advanced stage empyema could answer several fundamental questions: 1) which stages of empyema can best be treated with IPFT, 2) what the relationship between the “minimal effective dose” and disease severity is, and 3) is there one “minimal effective dose” that can be used for all patients with empyema or should the dose and dosing schedule be personalized for each patient.

## Bleeding Complications: Comparison of the Efficacy and Safety of tPA and uPA

Bleeding and treatment failure necessitating surgical intervention are the two major adverse events that have been reported when Alteplase IPFT is used alone or in combination with DNase (Froudarakis et al., 2008; Rahman et al., 2011; Piccolo et al., 2014; Alemán et al., 2015; Majid et al., 2016; Mehta et al., 2016; Jiang et al., 2020). Notably, the frequency of bleeding complications differs between clinical centers that are experienced with the MIST2 protocol (0–5.4%) (Rahman et al., 2011; Piccolo et al., 2014; Majid et al., 2016; Mehta et al., 2016), and those still trying to adopt it (up to 12–16%) (Alemán et al., 2015; Jiang et al., 2020), likely indicating the importance of procedural experience rather than the effect of dose of tPA (Skeete et al., 2004; Thommi et al., 2007). In contrast to the MIST2 protocol, treating patients (n = 48) with urokinase (two-chain, 100,000 IU daily for up to 6 days) demonstrated similar efficacy (97%) without bleeding complications (Alemán et al., 2015). Interestingly, urokinase appears to be more effective than tPA in patients with non-purulent complicated parapneumonic pleural effusions (Nie et al., 2014). Additionally, treatment with urokinase is associated with a lower likelihood of subsequent surgery when compared to IPFT with either tPA or streptokinase (Nie et al., 2014). Finally, direct comparison between tPA/DNase and urokinase has shown equal efficacy, albeit with a lower incidence of hemothorax with the latter (Bédat et al., 2019). Recent comparisons of IPFT with placebo have found that, while IPFT decreased the need for surgical intervention and overall failure of pleural fluid drainage, treatment with Alteplase versus urokinase was associated with a higher risk of bleeding (Altmann et al., 2019). The authors highlight the importance of considering disease severity when comparing different studies (Altmann et al., 2019). These observations agree with the results of a recent phase 1 trial using single chain urokinase (scuPA) (Beckert et al., 2019). It is not clear why urokinase and Alteplase demonstrate different efficacy in empyema in adults, while the former is successful in pediatric practice. Therefore, translational studies may help to understand whether urokinase is superior to tPA alone (Idell et al., 2007; Komissarov et al., 2013; Komissarov et al., 2016) or in combination with DNase, or if dose and choice of plasminogen activator should be personalized. Thus, clinical trials can be best informed by preclinical testing to define approaches to selecting treatment strategies that would account for disease severity as well as overall patient health and comorbidities. Targeting the mechanisms of a serpin, plasminogen activator inhibitor 1 (PAI-1), with monoclonal antibodies (mAbs) or a small Docking Site Peptide (DSP) resulted in an up to eight fold decrease in the effective bolus dose of plasminogen activator in rabbit models of chemically induced pleural injury and acute streptococcal empyema (Florova et al., 2015; Florova et al., 2018; Florova et al., 2021) without an increase in bleeding. Thus, PAI-1-targeted fibrinolytic therapy (PAI-1-TFT) aiming to support effective intrapleural fibrinolysis with low doses of tPA or uPA could become a novel approach to increasing the efficacy of treatment and decreasing the risk of bleeding complications in patients with empyema. Further preclinical studies of novel adjuncts and adjunct combinations for PAI-1-TFT would result in a novel, low-dose, well-tolerated treatment tractable in clinical settings.

## A Call for Novel Approaches to Assess the Likelihood of Success of IPFT

While patient mortality is negligible, the rate of IPFT failure in pediatric empyema remains around 15% (Sonnappa et al., 2006; St Peter et al., 2009; Livingston et al., 2016b; Long et al., 2016; Derderian et al., 2020). Defining reliable molecular predictors of treatment failure (Arnold et al., 2012020; Derderian et al., 2020; Khemasuwan et al., 2018; Livingston et al., 2016a) would therefore likely increase the efficacy of IPFT as a therapy for empyema in pediatrics. The striking difference in the efficacy of IPFT in adult and pediatric patients with empyema abrogates the possibility of applying successful pediatric treatments to adults. Moreover, biomarkers to identify patients in whom IPFT is likely to fail may not be equally successful in both populations. While clinicians acknowledge the importance of rapid diagnosis and treatment selection in advanced stage empyema, to the best of our knowledge there is currently no assay or procedure capable of predicting the likelihood of successful IPFT. Rapid diagnosis and staging of empyema and the choice of the most effective treatment could be critical for successful outcomes (Petrakis et al., 2010; Stefani et al., 2013; Reichert et al., 2017; Reichert and Bodner, 2018; Semenkovich et al., 2018). Prolonged delays between diagnosis and treatment may result in exacerbation of the empyema and necessitate more invasive treatment (Stefani et al., 2013).

IPFT activates endogenous plasminogen and is less effective in patients with plasminogen deficiency due to low expression, degradation, or imbalance in inhibitors. A hereditary or inflammation-induced plasminogen deficiency or overexpression of serpins (Mingers et al., 1999; Mehta and Shapiro, 2008; Martin-Fernandez et al., 2016; Prabhudesai et al., 2017; Saes et al., 2019; Tenbrock et al., 2019; Hangul et al., 2021) could affect the rate of fibrinolysis and thus outcomes of IPFT. Notably, while modern diagnostic approaches can evaluate the severity of empyema, they cannot assess the patient’s fibrinolytic system and determine whether IPFT could be a tractable approach. Unfortunately, neither Light’s Criteria (Light et al., 1972; Light, 1995; Light, 2013) nor current imaging diagnostic modalities–ultrasonography (Ramnath et al., 1998; Gates et al., 2004; Calder and Owens, 2009; Rahman et al., 2010) and chest CT (Kearney et al., 2000; Calder and Owens, 2009), can define optimal treatment and predict therapeutic outcomes (Kearney et al., 2000; Calder and Owens, 2009). Recent attempts to use artificial intelligence to predict the results of IPFT are still in an early stage (Khemasuwan et al., 2018; Ost, 2018; Khemasuwan et al., 2020). A RAPID score (Rahman et al., 2014) was developed to identify high-risk adults with empyema based on the degree of Renal impairment, Age exceeding 70 years, presence of Purulent pleural fluid, hospital-acquired Infection, and insufficient Diet (low albumin). Interestingly, patients with non-purulent pleural effusions had increased morbidity using this scoring system. This system can be used to stratify patient populations for comparability in clinical trial testing of different forms of IPFT or other interventions. A recent validation study demonstrated that the 3-months mortality increases from 2.3 to 29.3% with an increase in RAPID score from 0 to 2 to 5–7 (Corcoran et al., 2020). However, while high RAPID score is associated with higher 3-months mortality in adults (Corcoran et al., 2020; Touray et al., 1962018; White et al., 2015), it is not applicable for pediatric patients and does not identify patients that may be poor candidates for IPFT in any group. Identifying whether IPFT, VATS or other surgical options are the optimal treatment for a given patient remains challenging (Petrakis et al., 2004). Although a recently proposed algorithm focuses on surgical treatment for stage II and III empyema in adults (Reichert et al., 2017), the authors acknowledge a gap in our understanding regarding selection of optimal treatments and a lack of randomized, prospective clinical trials on catheter-directed IPFT versus minimally invasive surgery for such patients. Current biochemical tests and imaging techniques cannot assess the fibrinolytic system of a patient and inform on likely prognosis if they were offered IPFT or surgery, thus calling for the development of novel approaches to assist in treatment selection.

## A New Point of Care Test for Predicting the Outcome of IPFT

A novel biomarker, Fibrinolytic Potential (FP) was identified in preclinical studies and may address this issue (Florova et al., 2015; Komissarov et al., 2016; Idell et al., 2017; Florova et al., 2018; Beckert et al., 2019; Florova et al., 2021). Plasminogen accumulates in the pleural fluid of patients with empyema due to inhibited plasminogen activation. Consequently, fibrinolytic activity (FA_0_) in sampled pleural fluids increases dramatically with *ex vivo* supplementation with a plasminogen activator (FA_PA_), mimicking IPFT. Thus, FP, which is defined as the incremental change in fibrinolytic activity in pleural fluids at presentation induced by introducing an exogenous plasminogen activator (FA_PA_-FA_0_) accounts for much of the variability caused by individual parameters that affect fibrinolytic activity during IPFT, including levels of expression and degradation of plasminogen, other fibrinolytic proenzymes and enzymes, as well as inhibitors of fibrinolysis other than PAI-1. FP varies in humans by up to two orders of magnitude (Florova et al., 2015; Komissarov et al., 2016; Beckert et al., 2019). Low FP in pleural fluid—for example, due to low levels of expression or significant degradation of plasminogen, would predict IPFT failure in an individual (Beckert et al., 2019) and support surgery as the preferred intervention. FP is equally applicable to both adult and pediatric populations. Thus, the FP of pleural fluid sampled from a patient with empyema could become a prognostic biomarker to assist in informed decision making, with the value of FP associated with the probability of successful IPFT. Preclinical development and validation of a novel FP point-of-care test (POCT) that could differentiate between cohorts of patients that are candidates for IPFT (relatively high FP) and those in need of surgical intervention (low or undetectable FP), offers a paradigm shift in treatment of empyema. We posit that assessing FP can decrease the frequency of IPFT failure, morbidity and mortality, as well as overall cost of treatment for empyema, thereby becoming a critical element that contributes to decision making in personalized treatments. Confirmation awaits clinical trials, but the development of new diagnostics that can assess the suitability of a patient for IPFT is desirable and could potentially optimize therapy for individual patients with empyema.

## Patients With Advanced-Stage Empyema and Surgical Contraindications Contribute Dramatically to the Rates of Mortality and Treatment Failure

Surgery, whether VATS or pleural decortication, in advanced stage empyema (Bouros et al., 2002; Petrakis et al., 2004; Petrakis et al., 2010; Di Napoli et al., 2014; Majeed et al., 2021) could be considered a universal treatment option. VATS is a less invasive surgical approach to treat empyema in both adult and pediatric patients (Chambers et al., 2010; Petrakis et al., 2010; Scarci et al., 2011). Notably, the success rate (60–100%) and mortality (0–13%) of VATS for the treatment of stage II/III empyema has not changed over the last 20 years (Cassina et al., 1999). While studies of different approaches to managing parapneumonic effusions published 20 years ago indicated that surgery is associated with the lowest mortality (2%), followed by IPFT and VATS (4.5 and 5%, respectively) (Colice et al., 2000), they did not mention a dramatic difference between candidates for surgery and patients that have contraindications. There was a significant difference in the age between cohorts of patients that were treated surgically and those who were not. Multiple studies demonstrate that the age of patients could be considered an integral factor associated with contraindications to surgery, which is also related to mortality rates. Retrospective analysis of 4,424 patients (1987–2004) has shown a 2.8% annual rise in the incidence of empyema, with the fraction of patients requiring surgery increasing from 42.4 to 58.4%. Thirty-day mortality was higher in patients that were not treated surgically (16.6 versus 5.4%), and patients treated surgically were on average 10 years younger and with a lower comorbidity index (Farjah et al., 2007). For example, the incidence of empyema in Denmark (1997–2011; n = 6,874 (Søgaard et al., 2014)) increased by 26% for all patients, and by 87.3% for those older than 80 years. In that same timeframe, the mortality was 1.2% in 15 to 39-year-old patients and 20.2% in those older than 80 years of age. Thus, advanced age and comorbidities remain prognostic factors, and empyema continues to be a serious concern. Recent analysis of records of patients with empyema from the New York Inpatient Database (2009–2014; n = 4,095) that underwent chest tube drainage (n = 1,563), VATS and drainage (n = 1,313), and open decortication and drainage (n = 1,219) (Semenkovich et al., 2018), revealed higher mortality and readmission rates in the first group, suggesting that some of these patients would benefit from surgical intervention. However, the chest tube only cohort was significantly older than patients that underwent surgery (average 64 versus 56 and 57 years old; *p* < 0.001). Moreover, these patients had significantly higher (*p* < 0.001) rates of serious comorbidities such as congestive heart failure, septicemia, shock, coagulopathy, chronic anemia, cancer, as well as issues with pulmonary circulation, cardiac valve defects, peripheral vascular disease, chronic pulmonary disease, and renal disease. Thus, poorer outcomes in the first group were expected. While surgery is recommended when treating advanced stage empyema, there are several issues that affect morbidity and mortality when using a surgical approach. First, not every patient is fit for even minimally invasive surgery and thus, they undergo “non-surgical drainage” of pleural fluid. An example of such management could be found in the retrospective analysis of the survival of patients that underwent surgical and non-surgical approaches. The median age of patients referred to thoracoscopic surgery (n = 417) was almost a decade younger than those provided with non-surgical drainage (n = 185), 55.9 and 65.3 years, respectively (*p* < 0.001). Also, the non-surgical group had higher rates of comorbidities such as malignancy, malnutrition, liver cirrhosis and culture-positive pleural fluid (*p* ≤ 0.013). These indices clearly reflect the difference between patients with empyema that can and cannot be managed surgically, and could contribute to the higher mortality observed in the latter group (24.9 versus 7.5%; *p* = 0.001) (Chen et al., 2014). For patients with both advanced age and comorbidities who may be unwilling to undergo surgical intervention due to increased risks or contraindications, chest tube drainage remains the only option. This cohort of patients would benefit the most from the development of novel, well-tolerated, less invasive treatment approaches.

## Recent Approaches to Treat Empyema Patients With Surgical Contraindications

A small (n = 8) study of non-surgically treated patients with advanced stage empyema and a non-expandable lung, that is likewise associated with failure of IPFT (Huggins et al., 2018), reported success with slow (average 73.6 days) chest tube drainage (Biswas et al., 2016). Use of VATS without intubation (Kermenli et al., 2021), irrigation with saline, which was shown more effective than drainage alone (Hooper et al., 2015), and the use of an “extended MIST2 protocol” (tPA 10mg/DNase 5mg, twice daily; >6 doses; n = 20) in patients unable to receive surgery (McClune et al., 20162016) were used to address this problem. Notably, the “extended MIST2 protocol” demonstrated an increase in bleeding events (10% compared to 2.5%), and resulted in an improved protocol that requires further evaluation (Gilbert and Gorden, 2017). These clinical studies strongly call for translational research guiding the development of effective, less invasive treatments for patients with advanced stage empyema, who are unable to receive VATS or thoracoscopic surgery. Patients that decline or are unfit for surgical intervention could potentially benefit from treatment with low dose PAI-1-TFT, which allows for a decrease in the dose of plasminogen activator by almost an order of magnitude (Florova et al., 2015; Florova et al., 2018; Florova et al., 2021) or treatment with innovative encapsulated plasminogen activators in combination with ultrasound, which is successfully used in circulation (Bader et al., 2015; Klegerman et al., 2016; Klegerman, 2017; Miao et al., 2018). Any improvement in non-surgical drainage by decreasing the dose of the plasminogen activator, increasing the rate of intrapleural fibrinolysis, or increasing the half-life of plasminogen activating and fibrinolytic activities could positively affect the survival of this patient population. Even small improvements in the current treatment of advanced stage empyema could result increased efficacy and decreased mortality in this patient cohort.

## Validated Animal Models are Needed for Successful Preclinical Studies

Successful transition from bedside to bench heavily relies on the availability of validated animal models that recapitulate the disease in humans. The absence of such a model could result in unexpected adverse events or even treatment failure in human patients. While adequate models are critical for the development and testing of innovative treatments, there is a dearth of animal models that closely recapitulate advanced stage empyema in humans, indicating a gap in translational science that restricts our understanding of intrapleural fibrinolysis in advanced stage empyema. While mouse models of infectious pleural injury (Hsieh et al., 2008; Wilkosz et al., 2011; Tucker et al., 2016) are good for mechanistic studies of empyema in mice (Rovina et al., 2013; Lapidot et al., 2015; Tucker et al., 2016; Boren et al., 2017; Kamata et al., 2017; Tucker et al., 2019) they often lack correlation with human disease (Kanellakis et al., 2019) and are limited, as mouse fibrinolytic enzymes and inhibitors are structurally different from humans. Furthermore, murine fibrin structure differs significantly from that in humans (Pretorius et al., 2007; Jo et al., 2009; Dewilde et al., 2010). Rabbit models more closely recapitulate pleural injury and intrapleural fibrinolysis in humans (Idell et al., 2009; Komissarov et al., 2016; Kunz et al., 2004; Light et al., 1782000; Sasse et al., 1999; Sasse et al., 1997; Teixeira et al., 2000) and were used to develop the treatments assessed in the MIST2 clinical trial (Dikensoy et al., 2006; Zhu et al., 2006), which resulted in the current Alteplase/DNase protocol (Rahman et al., 2011). *Streptococcus pneumoniae*-induced pleural injury in rabbits (Komissarov et al., 2016) closely recapitulates the time course and major features of empyema observed in humans, including advanced stage disease, where standard IPFT becomes less effective. Moreover, the similarity of rabbit fibrin structure and intrapleural fibrinolytic system to humans (Pretorius et al., 2007) allows for testing of therapeutics that are used in clinical practice or intended for future clinical trials. Multiple loculations of fluid in the pleural space and pleural thickening with increasing severity of injury seen in rabbits are similar to what is observed during stage II and III empyema (Davies et al., 1999; Khemasuwan et al., 2018) in patients. Empyema in this rabbit model transitions from an acute to an advanced stage if untreated, and there is a notable decrease in the efficacy of IPFT in advanced stage empyema, as has likewise been observed in humans (Himelman and Callen, 1986; Temes et al., 1996). The decrease in the rate of intrapleural fibrinolysis in advanced stage empyema noted by clinicians (Thommi et al., 2007; Thommi et al., 2012; Thommi et al., 2014) contributes to the failure of IPFT. Compensating by increasing the dose of tPA increases the risk of bleeding complications. Over the last decade, the major mechanisms of intrapleural fibrinolysis identified in rabbit models of chemical and infectious pleural injury were also observed in humans (Komissarov et al., 2009; Komissarov et al., 2011; Karandashova et al., 2013; Komissarov et al., 2013; Florova et al., 2015; Komissarov et al., 2016; Florova et al., 2018; Idell and Rahman, 2018; Komissarov et al., 2018; Beckert et al., 2019). This resulted in identification and validation of PAI-1 as a molecular target (Karandashova et al., 2013) and development of PAI-1-TFT, which allows for an up to 8-fold decrease in the dose of plasminogen activator (Florova et al., 2015; Florova et al., 2018; Florova et al., 2021), as well as FP, a novel biomarker for assessing the likelihood of failure of IPFT in an individual patient (Florova et al., 2015; Komissarov et al., 2016; Idell et al., 2017; Florova et al., 2018; Beckert et al., 2019; Florova et al., 2021).

## Conclusion

The available literature indicates that at present, there is no one universal treatment for empyema in adult patients and opens new avenues for translational studies using validated animal models. The limits of IPFT could be determined using an appropriate animal model of chronic, advanced stage versus acute, early stage empyema with therapeutics such as human tPA, uPA, and DNase, which are already used in clinical practice. Newer options, such as low dose PAI-1-TFT or plasminogen activators encapsulated in liposomal carriers could likewise be tested in preclinical studies in order to identify and develop innovative treatments for empyema and expanding treatment to patients, who currently are not candidates for IPFT. Varying the plasminogen activator dose, dosing frequency and schedule, as well as exploring novel adjuncts that could increase efficacy, could inform the design of more personalized approaches to the treatment of advanced stage empyema in humans. We believe that it could be beneficial to use biomarker analyses of pleural fluid to develop POC tests that inform on which treatment is the most beneficial for a given patient. Simultaneously, low-dose, well-tolerated treatments for advanced stage empyema could expand the cohort of patients, including those that are of advanced age and with multiple comorbidities, that could benefit from IPFT. Preclinical development and testing of new methods of predicting patient prognosis after intervention and targeted treatments hold promise to improve outcomes in patients with organizing pleural injury with failed drainage and poor responses to currently available therapy.
